# Cytological characteristics of blueberry fruit development

**DOI:** 10.1186/s12870-024-04809-y

**Published:** 2024-03-12

**Authors:** Xianqin Wan, Zewei Wu, Dongchan Sun, Li Long, Qiling Song, Chao Gao

**Affiliations:** Institute for Forest Resources and Environment of Guizhou, Key laboratory of forest cultivation in plateau mountain of Guizhou province, College of Forestry, Guizhou University, Guiyang, 550025 China

**Keywords:** Blueberry, Powderblue, Blueberry, Fruit size, Fruit development, Seed development, Anatomic observe

## Abstract

Using the blueberry cultivar "Powderblue" after pollination, fruits at different developmental stages were collected for study. The transverse and longitudinal diameters, individual fruit weight, and fruit water content were measured during their development. Employing tissue sectioning and microscopy techniques, we systematically studied the morphological features and anatomical structures of the fruits and seeds at various developmental stages, aiming to elucidate the cytological patterns during blueberry fruit development. The results of our study revealed that the "Powderblue" blueberry fruit growth and development followed a double "S" curve. Mature "Powderblue" blueberries were blue-black in color, elliptical in shape, with five locules, an inferior ovary, and an average fruit weight of 1.73 ± 0.17 g, and a moisture content of 78.865 ± 0.9%. Blueberry fruit flesh cells were densely arranged with no apparent intercellular spaces, and mesocarp cells accounted for 52.06 ± 7.4% of fruit cells. In the early fruit development stages, the fruit flesh cells were rapidly dividing, significantly increasing in number but without greatly affecting the fruit's morphological characteristics. During the later stages of fruit development, the expansion of the fruit flesh cells became prominent, resulting in a noticeable increase in the fruit's dimensions. Except for the epidermal cells, cells in all fruit tissues showed varying degrees of rupture as fruit development progressed, with the extent of cell rupture increasing, becoming increasingly apparent as the fruit gradually softened. Additionally, numerous brachysclereids (stone cells) appeared in the fruit flesh cells. Stone cells are mostly present individually in the fruit flesh tissue, while in the placental tissue, they often group together. The "Powderblue" blueberry seeds were light brown, 4.13 ± 0.42 mm long, 2.2 ± 0.14 mm wide, with each fruit containing 50–60 seeds. The "Powderblue" seeds mainly consisted of the seed coat, endosperm, and embryo. The embryo was located at the chalazal end in the center of the endosperm and was spatially separated. The endosperm, occupying the vast majority of the seed volume, comprised both the chalazal and outer endosperm, and the endosperm developed and matured before the embryo. As the seed developed, the seed coat was gradually lignified and consisted of palisade-like stone cells externally and epidermal layer cells internally.

## Introduction

Blueberries (*Vaccinium spp*.) are a member of the Ericaceae family and the genus *Vaccinium*, originally native to North America. They are widely distributed across the temperate and subtropical regions of the Northern Hemisphere [1]. Approximately 400 species of the genus *Vaccinium* have been identified globally, with about 91 species and 28 varieties found in China, primarily in the northeastern and southwestern regions. Blueberries are categorized into three main types: rabbiteye, highbush, and lowbush blueberries [2]. The United Nations Food and Agriculture Organization lists blueberries as one of the five healthiest human foods, earning them the title of "King of Fruits Worldwide" [3]. As of 2020, the number of countries and regions cultivating blueberries worldwide increased from 58 in 2016 to 71. The total cultivation area grew by 55.15% compared to 2016, reaching 205,670 hectares. The total yield doubled from 466,800 tons in 2012, currently reaching 1,387,700 tons [4].

The blueberry fruit development includes different stages and processes such as cell division, enlargement, and the transformation and accumulation of assimilates [5, 6]. It is the cornerstone to the attainment of desirable fruit quality, anthocyanin accumulation in the fruit, and the postharvest longevity of the mature fruit. From a cytological perspective, the fruit cell number, their size, and the extent of intercellular spaces are vital factors determining fruit size and shape. After the fruit set, the continuous cell division and expansion in the fruit flesh manifest as dynamic changes in fruit size [7]. Cell division during fruit development primarily occurs after pollination and the initial developmental stages of the young fruits, which determines the number of cells in the fruit, subsequently influencing its final volume. Cell expansion depends on cell wall relaxation and turgor pressure [8] and mainly occurs in the middle and later fruit development stages. No absolute temporal boundaries have been identified between cell division and cell expansion [9]. The blueberry fruit's morphological characteristics directly affect its market value and economic benefits. Different blueberry varieties have distinct fruit morphologies, with the blueberry fruit color being primarily regulated by the accumulation of anthocyanins [10, 11]. The growth curve of the fruit describes the dynamic changes in fruit development and corresponds to the growth pattern of the fruit after ovary fertilization. Extensive research has indicated that blueberry fruits follow a double "S" growth curve [12]. Although the mature blueberry fruit consists mostly of flesh, and seeds occupy a smaller proportion, seed development is a crucial phase in the life cycle of higher plants. It can directly impact fruit properties and is vital for plant reproduction and crop yield [13].

At present, research on blueberries has primarily focused on the selection and development of superior varieties [14], anthocyanins biosynthesis and regulation of their accumulation [15], fruit postharvest longevity [16], and the quality of mature fruits [17]. Studies related to blueberry fruit growth are mostly centered around fruit development and morphological changes. On the other hand, systematic studies on cell division, enlargement, and expansion are scarcely reported. To this end, using the "Powderblue" blueberry variety, we comprehensively investigated fruit flesh cell division and expansion patterns during fruit development at the cellular level, providing a cytological basis for understanding blueberry fruit development and morphological change patterns.

## Materials and methods

### Experimental site and plant materials

The experiment was conducted at the Shengfeng Organic Blueberry Base located in Meiman Village, Xiajiang Town, Congjiang County, Qiandongnan Prefecture, Guizhou Province, China (25°80′N, 108°76′E) at an altitude of 523.98 m. The location is characterized by a warm subtropical climate with four distinct seasons, with no extreme heat in summer and no severe cold in winter. The annual average temperature is 18.5 °C, with a yearly average rainfall of 1185.9 mm. The average frost-free period extends for 328 days each year, with an average annual sunshine duration of 1304.9 h and an average annual evaporation of 1185 mm. For this study, fruits from the blueberry cultivar "Powderblue", which were well-grown and developed within the blueberry farm, were selected as the experimental materials.

### Morphological characteristics and water content of blueberry fruits

Three robust blueberry plants with similar vigor were selected for tagging and registration. Starting from the day of registration, every three days, 30 medium-sized blueberry fruits were collected from five directions—east, south, west, north, and center—at the mid-level of the canopy for morphological characteristic measurements.

The transverse and longitudinal diameters and the calyx diameter of the fruits were measured with a vernier caliper. The fruit shape index was calculated as the fruit's longitudinal diameter divided by the fruit's transverse diameter. The classification was based on Han's fruit shape standards: an index from 0.6 to 0.8 indicated a flat-round shape, > 0.8 to 0.9 indicated a round or nearly round shape, > 0.9 to 1.0 indicated an oval or conical shape. Values above 1.0 indicated an elongated shape.

A precision analytical balance was used to determine the fresh weight of individual fruits. The fruits were then dried to a constant weight in an oven to determine the dry fruit weight. Changes in the fruit water content during development were analyzed and calculated. At the same time, photographs were taken to record the changes in fruit color throughout the developmental process.

### Characteristics of cells during blueberry fruit development

The characteristics of blueberry fruit cells were studied using the conventional paraffin sectioning method. The material was placed in Carnoy's fluid for vacuum infiltration, then transferred to 70% alcohol and stored at 4 °C in a refrigerator. During the experiment, the material was cut into 3–5 mm thin slices,stained with hematoxylin for 1 day, and then washed with water and then subjected to a graded alcohol series from low to high concentrations, followed by treatment with alcohol and xylene. The material was then embedded in paraffin at different temperature conditions. Sections were made using a paraffin microtome (Leica RM2235, Germany), then mounted, dried, dewaxed, sealed, and dried again (Gao C, 2019). Photographs were taken, and observations were recorded under a biological microscope (OLYMPUS BX53 (LED), Japan).

### Changes in cell number and cell size during the development of blueberry fruits

The changes in cell number and cell size during the development of blueberry fruits were analyzed using the counting method of Renaudin [18]. Cross-sectional paraffin sections of six tissues of the blueberry fruits were prepared: from the epidermis, outer mesocarp, mesocarp, inner mesocarp, central mesocarp, and placenta. The number of cells in each tissue was counted. The cell size in each part was calculated using cellSens Standard software. Data analysis and graphing were performed using Excel 2016 and Origin 2022 software.

### Cytological characteristics of blueberry seed development

Tweezers were used to extract the seeds from the fruits at different developmental stages, which were then observed and photographed under a stereomicroscope (Leica KL300 LED, Germany) to identify changes in their external morphological characteristics. The conventional paraffin sectioning method was used to observe the cytological features during seed development, following the sample preparation protocol described in section1.3.

## Results and analysis

### Characteristics of blueberry fruit development

**Fig. 1:**
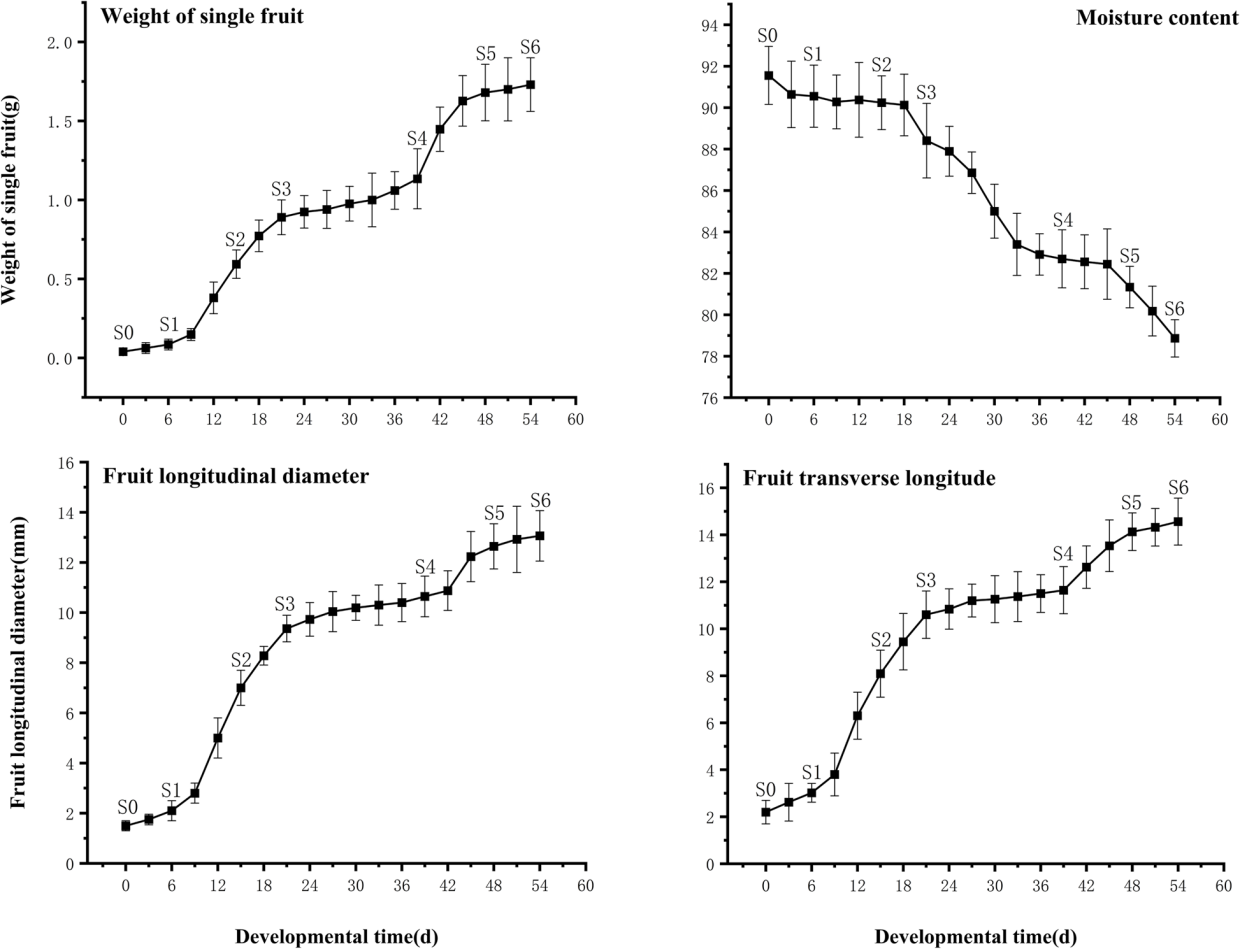
Evolution of single fruit weight, transverse diameter, longitudinal diameter, and water content during blueberry fruit development

Based on the growth indices of blueberry fruits (Fig. 1), two rapid growth periods occur during the blueberry fruit development, corresponding to a double "S"-shaped growth curve. In the two rapid fruit growth periods, the weight of individual fruits increased rapidly, with the rate of weight increase in the second rapid growth period being greater than in the first. During the blueberry fruit development, its transverse and longitudinal diameters expanded in certain proportions, which also corresponded to a double "S"-shaped curve, with the growth rate during the first rapid growth period exceeding that of the second. However, the fruit shape index showed a declining trend. Moreover, throughout fruit development, the water content of the fruits exhibited a downward trend.

**Fig. 2:**
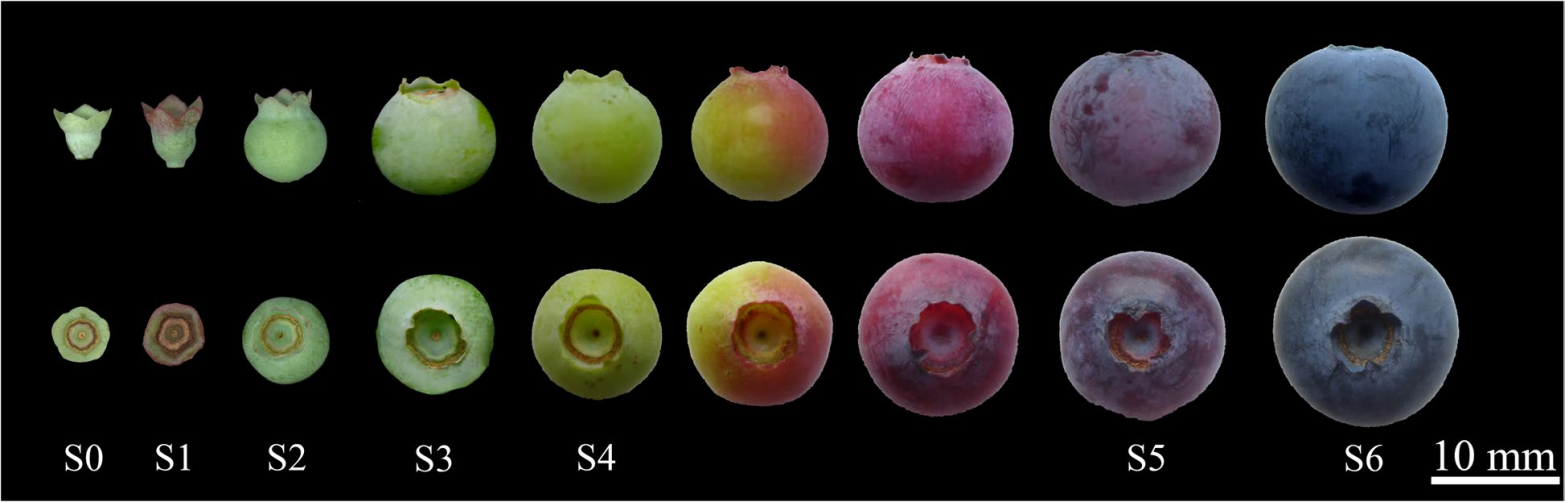
Dynamics of blueberry fruit growth and development

Based on the growth curve trends of single fruit weight, transverse diameter, and longitudinal diameter, the development of blueberry fruits was divided into seven main stages (Fig. 2), labeled as Stage 0 to Stage 6 (abbreviated as S0-S6). The S0-S1 period lasted about 6 days, during which the fruit grew relatively slowly, with an average daily weight increase of only 0.0072 g/day. The S0 stage corresponded to the initial fruit developmental phase, with fruits being cone-like in shape and green in color, with a single fruit weight of 0.039 ± 0.02 g. The S1 stage marked the initiation of the first rapid growth phase, during which the lower part of the ovary started to expand, the fruit remained green in color, and the single fruit weight increased to 0.0847 ± 0.034 g, more than doubling compared to S0. The S1-S3 period lasted about 15 days, with the fruit growing rapidly, with an average daily weight increase of 0.0539 g/day. The S2 stage corresponded to the midpoint of the rapid growth period, with fruit shape transitioning from cone-like to spherical, still green in color. The S3 stage marked the end of the first rapid growth period and the start of a fruit growth stagnation period, with the fruit being still green and the single fruit weight reaching 0.89 ± 0.11 g. The fruit growth then entered a stagnation phase, the S3-S4 period. The initiation of the S4 stage marked the end of the growth stagnation period and the start of the second rapid growth period, with the fruit color turning yellow. The S3-S4 period lasted between 8 to 12 days. During the S4 stage, the development of the fruit decelerated, with the fruit color gradually changing from green to yellow and an average daily weight increase of 0.0222 g/day. The S5 stage marked the end of the second rapid growth period, with the fruit color turning purple-red and the single fruit weight reaching 1.68 ± 0.179 g. The S4-S5 period corresponded to the second rapid growth phase of fruit development, with a duration of 8 to 10 days, with the fruit color changing from yellow to red and finally to purple-red. The average daily weight increase was 0.0380 g. Due to the rapid change in fruit color during the S4-S5 period, sampling later in this phase was not done based on color. The S6 stage represented the end of the color change period, with the fruit color shifting to blue-black, indicating that the fruit has matured. The single fruit weight reached 1.7 ± 0.2 g,. The mature blueberry fruits had a shape index of 0.91, corresponding to an oval shape. The calyx lobes were erect, showing a semi-open or open type of calyx with five calyx segments.

### Anatomical features of blueberry fruit cells during fruit development

**Fig. 3:**
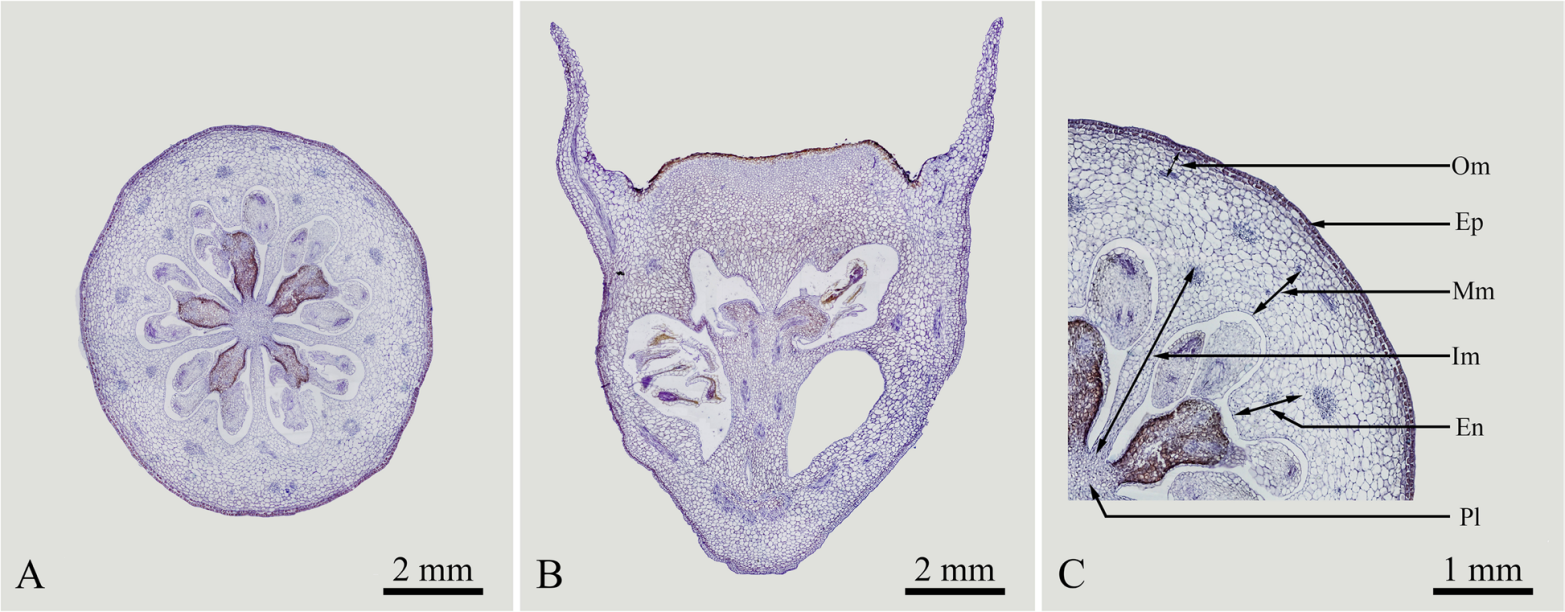
Paraffin sections of blueberry fruits at the S0 stage. **A** Transverse section of the fruit; **B** Longitudinal section of the fruit; **C** Enlarged partial transverse section of the fruit. Ep:Epidermis; En:Endocarp; Im:Inner mesocarp; Mm:Middle mesocarp; Om:Outer mesocarp; Pl:Placental tissue

Paraffin sections of fruits in the S0 stage indicated that "Powderblue" blueberry fruits had five carpels and five locules, with an inferior ovary and a central axial placenta. The ovules were mostly arranged in double rows (Fig. 3).

#### Anatomical features and development of epidermal cells and outer mesocarp cells

**Fig. 4:**
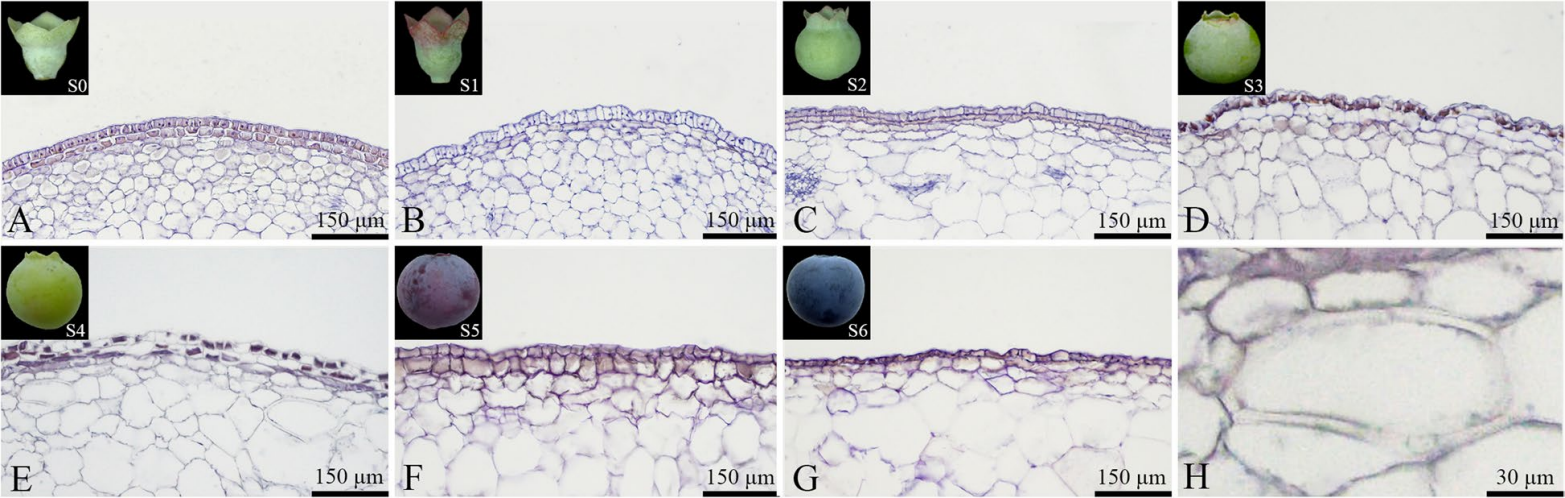
Developmental dynamics of epidermal and outer mesocarp cells in blueberry fruit. **A**-**G** Cross-sectional paraffin sections of the epidermis and outer mesocarp; **H** Stone cells within the outer mesocarp

The epidermal cells of the "Powderblue" blueberry fruit were rectangular, uniform in size, and tightly arranged. The epidermal cells did not show signs of rupture throughout fruit growth and development, while no stone cells were present (Fig. 4). The outer mesocarp cells of the blueberry fruit were irregularly shaped, mostly near-circular (Fig. 4). By the S4 stage, a small number of stone cells appeared within the outer mesocarp, with intact thin-walled cell structures (Fig. 4E and H). By the S5 stage, the number of stone cells in the outer mesocarp increased, and a few thin-walled cells began to rupture (Fig. 4F). By the S6 stage, the number of stone cells in the outer mesocarp continued to increase, and many thin-walled cells ruptured and dissolved, losing their intact structure (Fig. 4G).

**Fig. 5:**
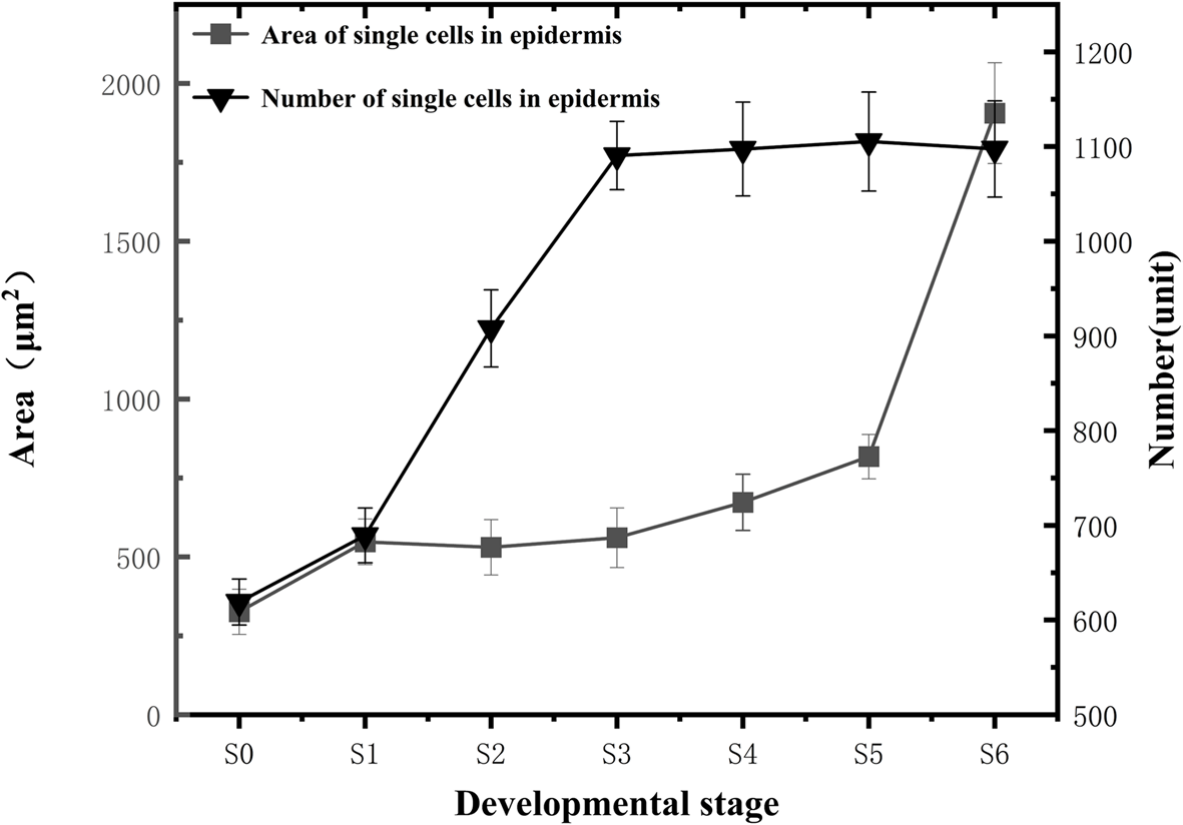
Changes in the number and size of epidermal cells

An increasing trend was generally observed for the total number of epidermal cells during the blueberry fruit growth and development (Fig. 5). From the S0 to S1 stage, the increase in the total epidermal cell count was relatively slow. From S1 to S3, epidermal cells proliferated rapidly, and their numbers sharply increased, peaking at (1090.59 ± 36) cells. From S3 to S6, the number of epidermal cells ceased to increase and remained stable, with the primary difference being an increase in individual cell size. Throughout fruit development, the size of individual epidermal cells generally increased. However, from S0 to S5, the increase in individual cell size was slow. The significant increase in individual cell size primarily occurred during the S5 to S6 stage, when the color transition was completed and the fruit matured. During this stage, epidermal cells became enlarged and changed into elongated rectangles, with an observed individual cell area of (1905.86 ± 159.56) μm^2^.

**Fig. 6:**
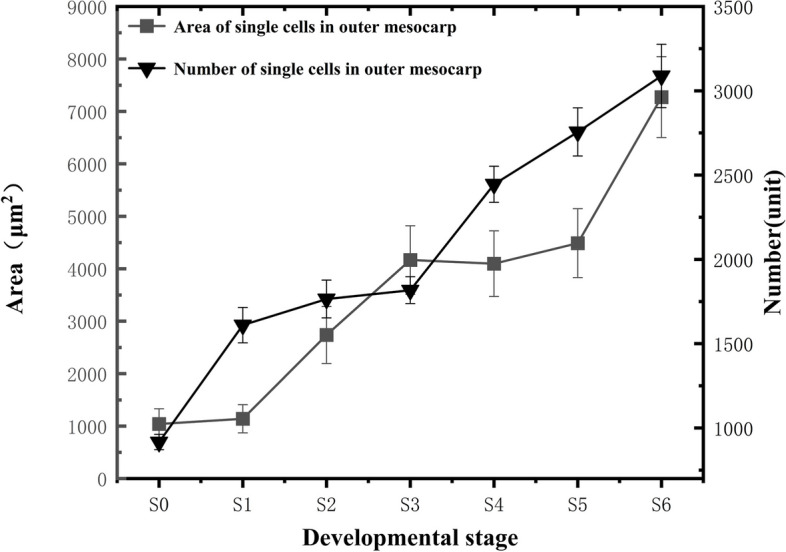
Changes in the number and size of outer mesocarp cells

The size of the outer mesocarp cells generally tended to increase during blueberry fruit growth and development (Fig. 6). During the S0-S1 stage, there was no significant change in the measured area of individual cells, but the number of cells increased sharply. It was followed by the first rapid cell enlargement stage, the S1-S3 period, during which the measured area of individual cells rapidly increased. The S3-S5 stage period was characterized by a slower cell enlargement, after which the fruit entered the second rapid cell enlargement stage, the S5-S6 period. The rate of cell size increase in the S5-S6 period stage was greater than that of the S1-S3 period. The number of outer mesocarp cells showed a general increasing trend in line with the progression of fruit growth and development, with different rates of increase in each stage. Specifically, the rate of increase during the early fruit development stage, S0-S3, was lower than during the later development stage, S3-S6.

#### Anatomical features and development of mesocarp cells

**Fig. 7:**
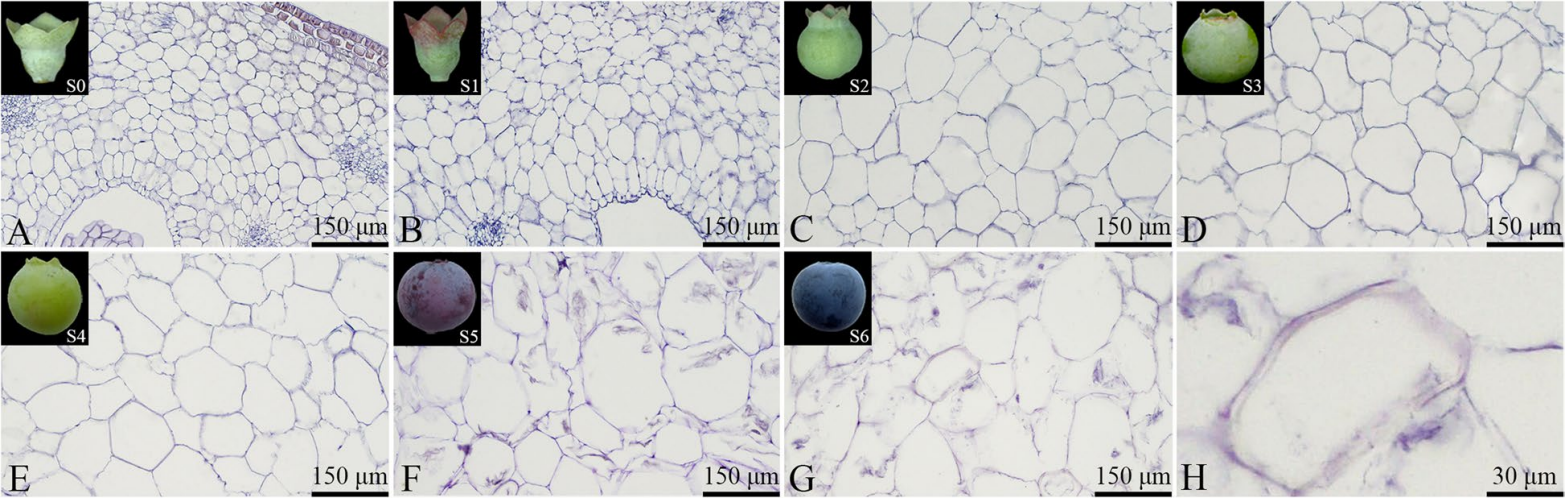
Developmental dynamics of mesocarp cells in blueberry fruit. **A**-**G** Cross-sectional paraffin sections of the mesocarp; **H** Stone cells within the mesocarp

Mesocarp cells were irregular in shape, predominantly near-circular, and tightly arranged (Fig. 7). During the S0-S1 stage, the mesocarp cells maintained a uniform structure, and no stone cells were present (Figs. 7A and B). With the progression of fruit growth and development, by the S4 stage, mesocarp cells began to exhibit a limited occurrence of cell rupture but largely retained their intact structure (Fig. 7C to E). In the S5-S6 stage, as numerous mesocarp cells dissolved and ruptured, the mesocarp cells almost entirely lost their structural integrity, and a large number of stone cells was observed (Figs. 7F and H).

**Fig. 8:**
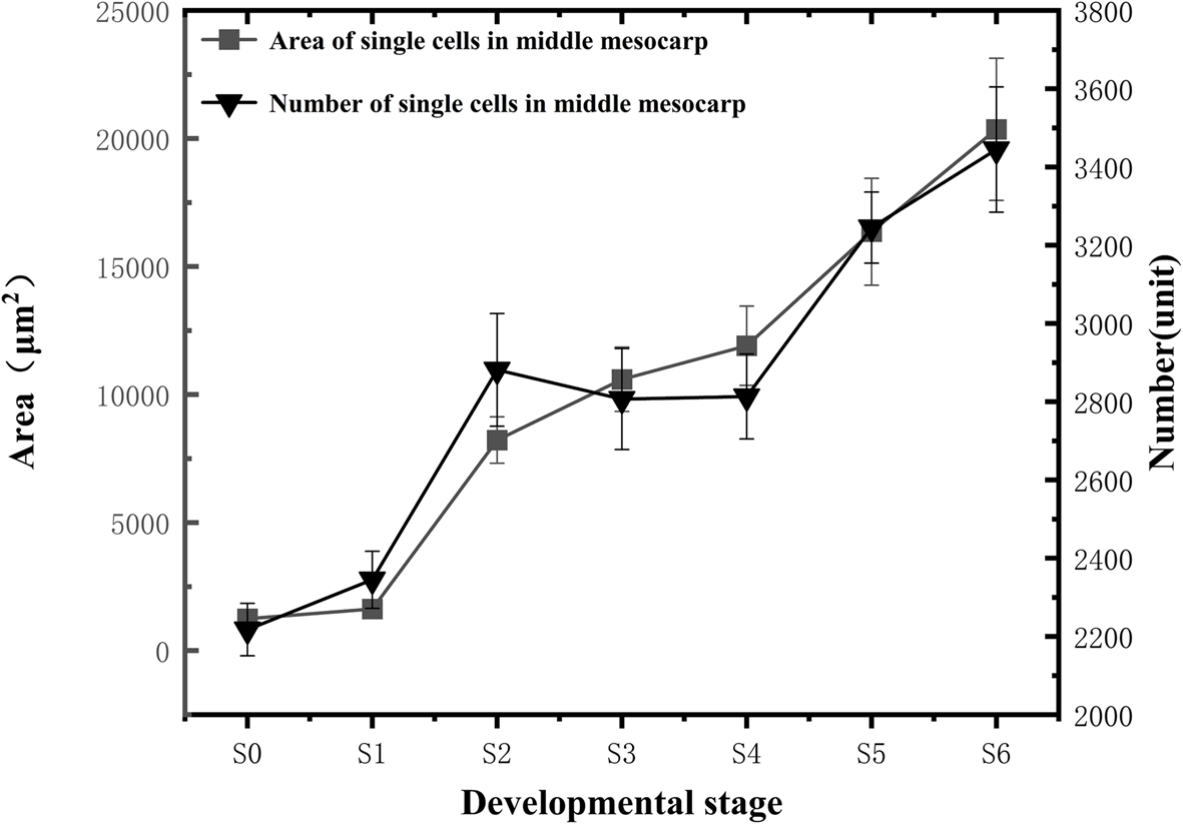
Changes in the Number and Size of Mesocarp Cells

The size of mesocarp cells generally exhibited an increasing trend during fruit development, characterized by two rapid enlargement stages (Fig. 8). During the S0-S1 stages, the increase in the measured area of individual cells was low. The first rapid cell enlargement stage occurred from S1 to S3, followed by a gradual enlargement rate deceleration from S3 to S4. The second rapid cell enlargement stage occurred from S4 to S6, with the mature fruit's mesocarp cells reaching an individual cell area of 20,361 ± 2774.45 μm^2^. The number of mesocarp cells also shows an increasing trend throughout fruit development, with two periods of rapid increase (Fig. 8). Their number increased slowly during the S0-S1 stage, then entered the first rapid increase period from S1 to S2, with cells rapidly proliferating and their number sharply increasing. During this period, the fastest rate of cell number increase was observed, at approximately 59.6 cells/day. The S3-S4 stage marked a stagnation period with little change in cell number. The second rapid cell number increase stage occurred from S4 to S6, with the mature blueberry mesocarp cell count reaching 3444.7 ± 160.3 cells.

#### Anatomical features and development of the inner mesocarp cells

**Fig. 9:**
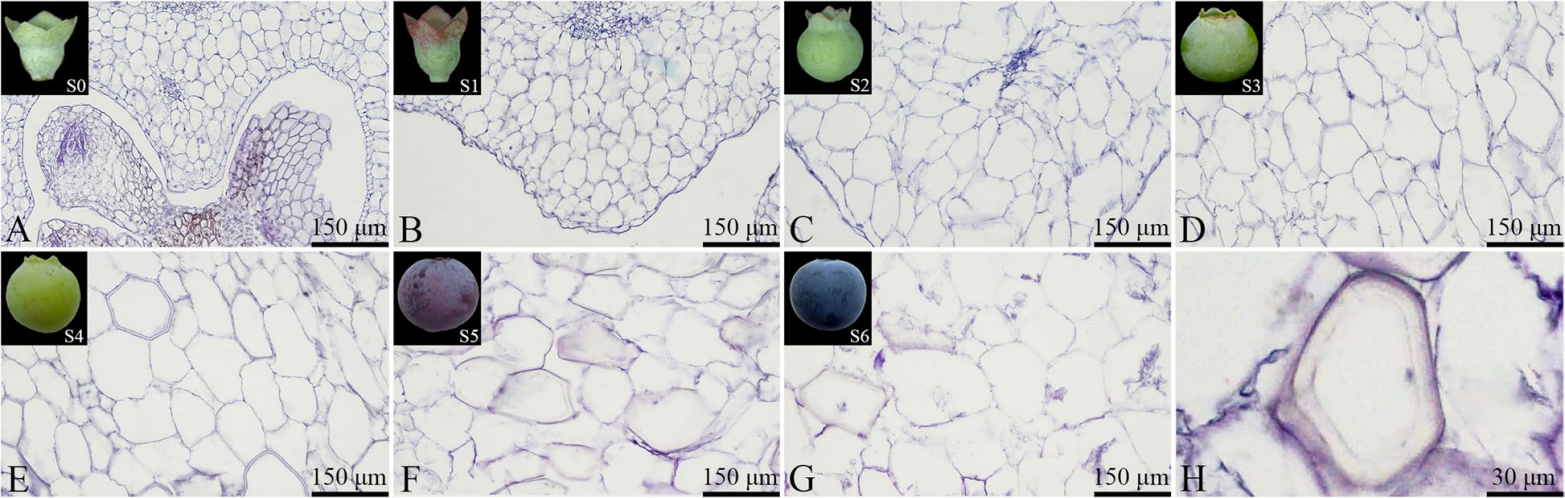
Developmental dynamics of inner mesocarp cells in blueberry fruit. **A**-**G** Cross-sectional paraffin sections of the inner mesocarp; **H** Stone cells within the inner mesocarp

**Fig. 10:**
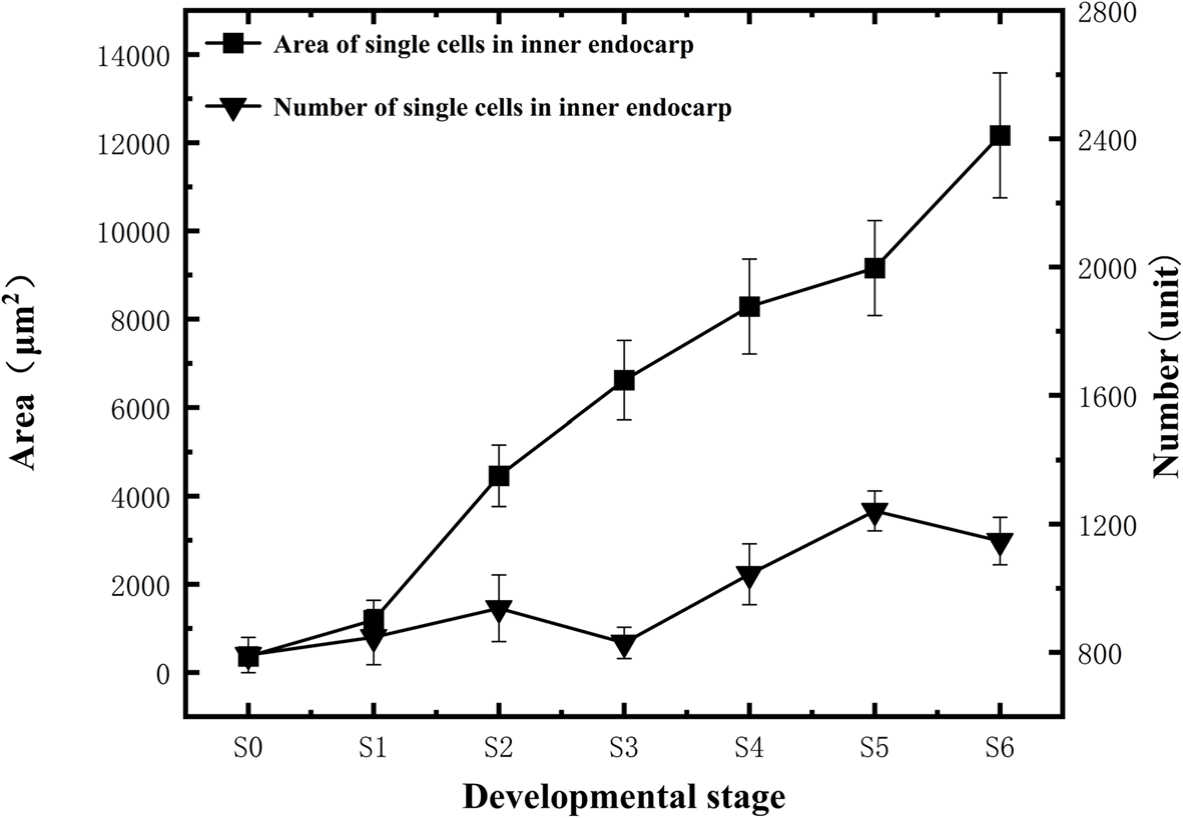
Changes in the number and size of inner mesocarp cells

The inner mesocarp cells were irregularly shaped (Fig. 9). During the S0-S1 stage, the inner mesocarp cells maintained a complete structure and were tightly arranged (Figs. 9A to B). The cells predominantly elongated longitudinally from the S1 to the S4 stage (Figs. 9A to E). At the S4 stage, slight rupturing and dissolution of the inner mesocarp cells was observed, along with the appearance of a few stone cells (Fig. 9E). From S4 to S6, the cells were mainly laterally elongated, and the extent of cell rupturing and dissolution increased. The mature fruit's inner mesocarp cells could no longer maintain their complete structure, and the number of stone cells was similarly increased (Figs. 9E to H). Both the measured area of individual inner mesocarp cells and the total number of cells showed an increasing trend throughout fruit development (Fig. 10), reaching 12,167.8 ± 1415.84 μm^2^ and 1147.36 ± 74.8 cells during fruit maturity, respectively.

#### Anatomical features development of endocarp cells

**Fig. 11:**
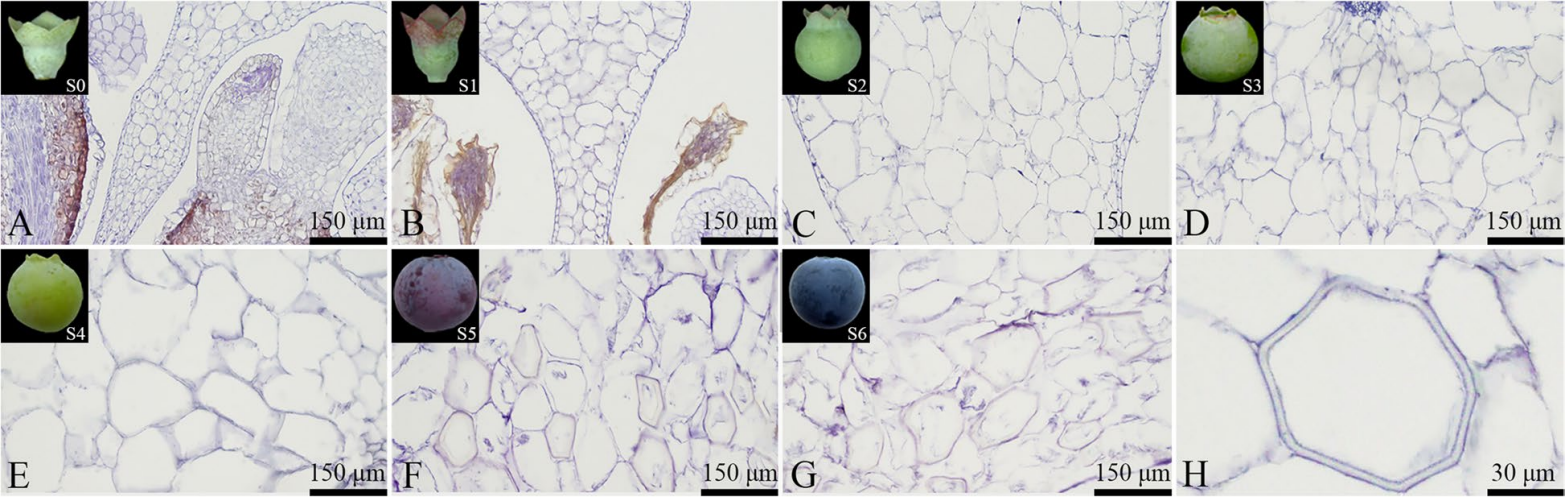
Developmental dynamics of endocarp cells in blueberry fruit. **A**-**G** Cross-sectional paraffin sections of the endocarp; **H** Stone cells within the endocarp

In the S0-S1 stage, the endocarp cells were relatively regular in shape (Fig. 11A to B). As the fruit development progressed, their cell shape became increasingly irregular. During the S0-S1 stage, the endocarp cells maintained a complete structure, and no stone cells were present (Fig. 11A to B). In the S2-S4 stage, the main manifestation is the increase of the area of individual cells (Fig. 11C to E). As the fruit continued to develop, by the S4 stage, a very small number of endocarp cells started to rupture, though they largely retained their intact structure. In the S5-S6 stage, as numerous endocarp cells underwent dissolution and rupture, the endocarp cells could no longer maintain their complete structure, and many stone cells emerged (Figs. 11F to H).

**Fig. 12:**
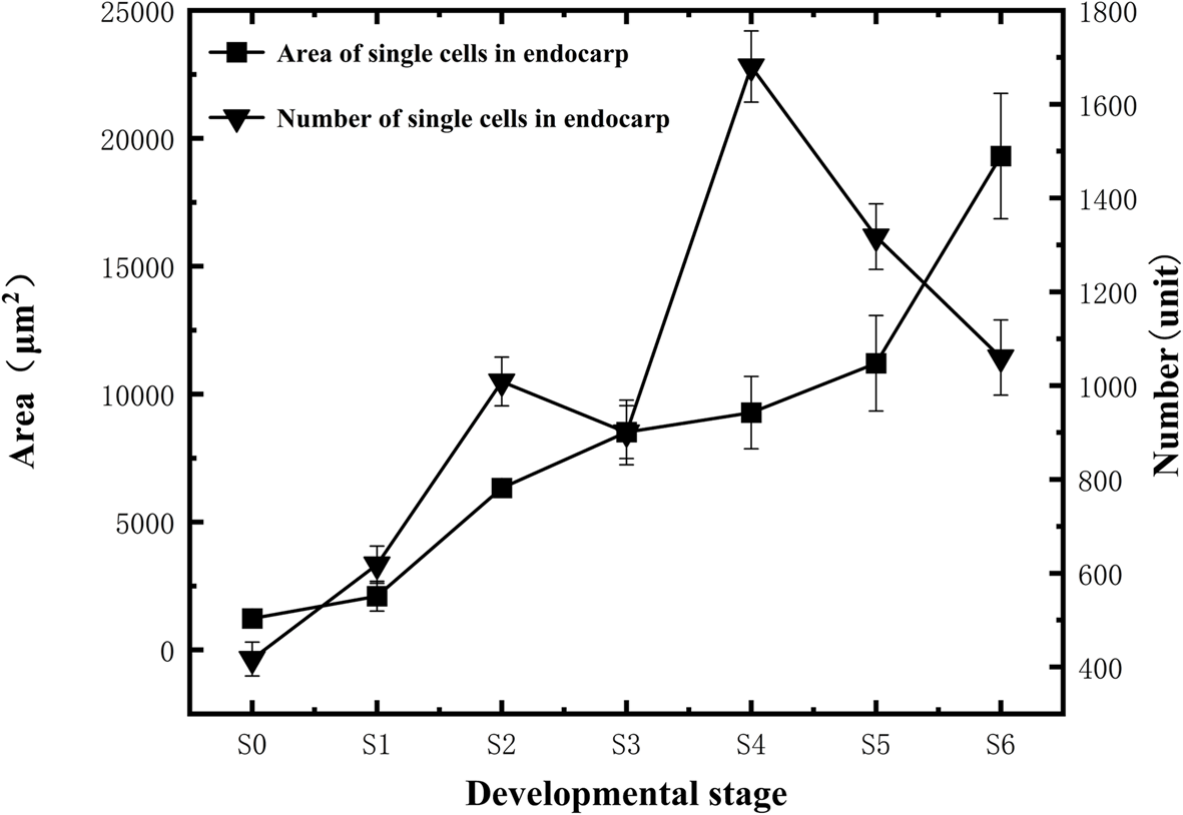
Changes in the number and size of endocarp cells

The endocarp cell size generally increased with the fruit's development, with varying growth rates at each stage, reaching a measured area of 19,307.80 ± 2448.39 μm^2^ in the mature fruit endocarp (Fig. 12). The total number of endocarp cells showed an increasing trend, which varied throughout fruit development. From S0 to S2, an increasing trend in endocarp cell number was observed. On the other hand, from S2 to S3, the cell count exhibited a decreasing trend. In the S3 to S4 stage, the cells proliferated rapidly, and their number sharply increased, with the fastest cell number increase rate during this period measured at approximately 43.34 cells/day. From S4 to S6, there was a sharp decrease in cell count, with mature blueberry endocarp cells being 1020.70 ± 80.00 in total (Fig. 12).

#### Anatomical features and development of placental cells

**Fig. 13:**
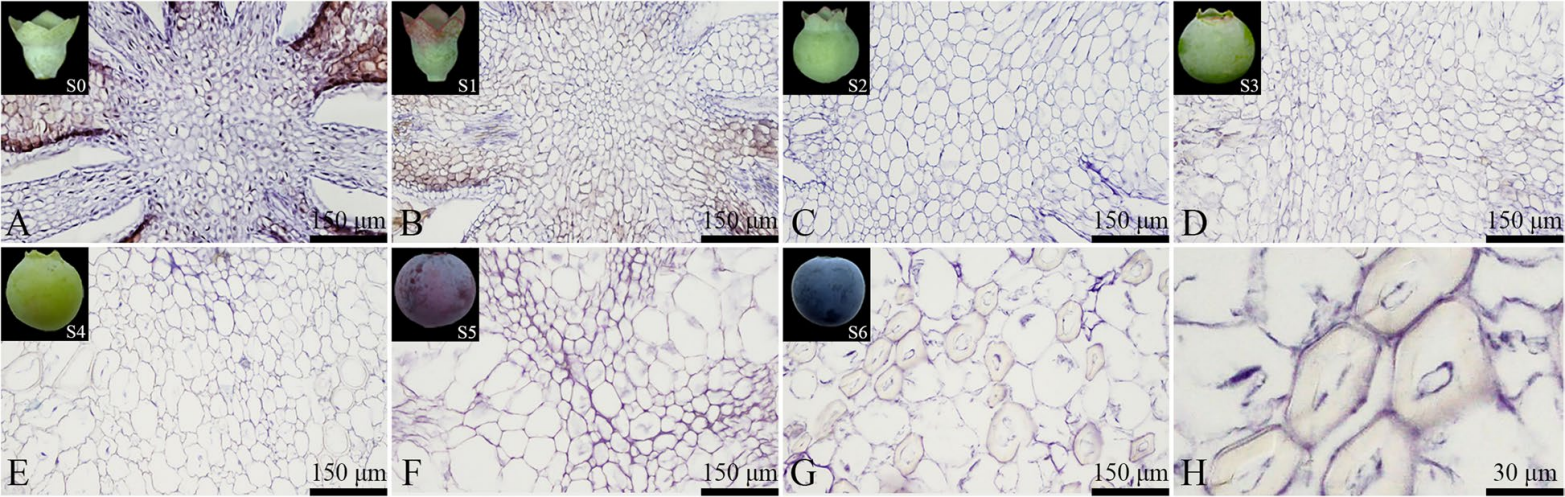
Developmental dynamics of placental cells in blueberry fruit. **A**-**G** Cross-sectional paraffin sections of the placental cells; **H** Stone cells within the placenta

Placental cells were uniform in shape and size and tightly arranged (Fig. 13). In the later stage(S5-S6 period) of fruit growth, a small number of placental cells exhibited rupture and dissolution, and a large number of stone cells appeared within the mature fruit's placenta (Figs. 13F to H).

**Fig. 14:**
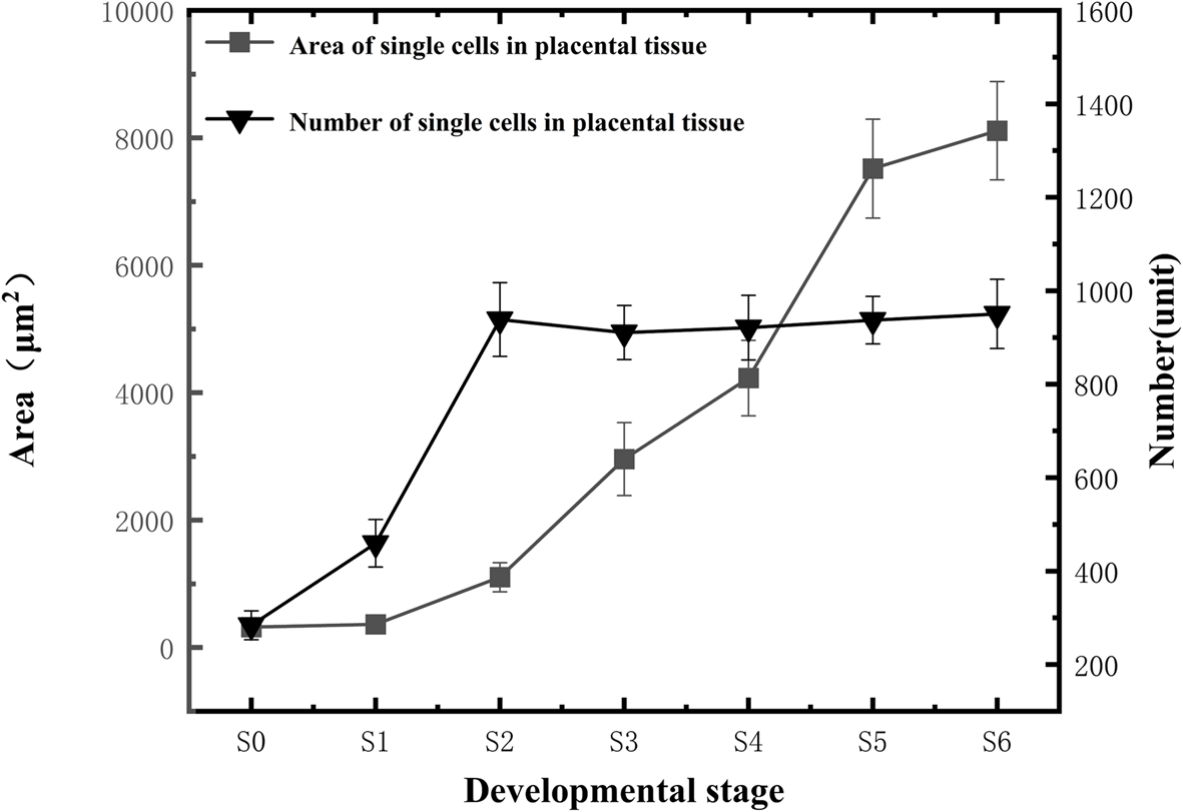
Changes in the number and size of placental cells

As the fruit developed, the size of placental cells showed an increasing trend, albeit with varying rates of increase at each developmental stage (Fig. 14). The measured area increase rate was fastest during the S4-S5 stage, reaching approximately 365.1822 μm^2^/day. The second fastest rate of increase was observed at the S2-S3 stage, with a rate of 309.055 μm^2^/day. The number of placental cells primarily increased during the S0-S2 stage, and the cell count remained relatively stable from the S3 to S6 stage, with the total number of mature placenta cells reaching 950.344 ± 74.4.

### Vascular bundle development

#### Development of carpelarry central vascular bundles

**Fig. 15:**
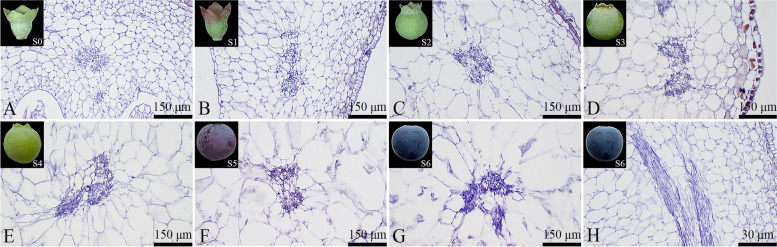
Developmental dynamics of carpelarry central vascular bundles in blueberry fruit. **A**-**G** Cross-sectional paraffin sections of the carpellary central vascular bundles; **H** Longitudinal paraffin section of a mature carpellary central vascular bundle

In blueberry fruits, the carpellar central vascular bundles comprise 2–3 vascular bundles, forming a vascular bundle group. As the fruit developed, the cross-sectional area of the carpellary central vascular bundles increased, reaching its largest area during the fruit maturation. Throughout their development, the carpellary central vascular bundles maintained structural integrity (Fig. 15).

#### Development of carpellary lateral vascular bundles

**Fig. 16:**
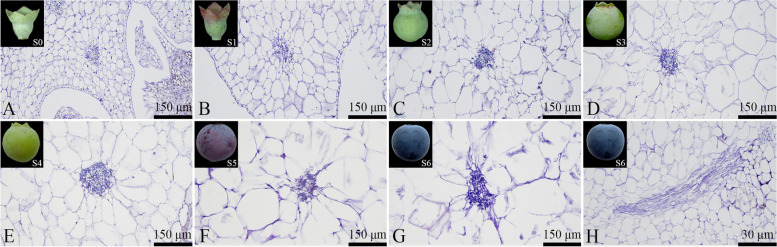
Developmental dynamics of carpellary lateral vascular bundles in blueberry fruit. **A**-**G** Cross-sectional paraffin sections of the carpellary lateral vascular bundles; **H** Longitudinal paraffin section of a mature carpellary lateral vascular bundle

The carpellary lateral vascular bundles in blueberries are branches of the main vascular bundles. As the fruit development progressed, the cross-sectional area of the carpellary lateral vascular bundles increased, reaching its largest area during the fruit maturation stage (Fig. 16). During the S0-S4 stages of blueberry fruit development, the carpellary lateral vascular bundles appeared nearly circular (Figs. 16A to E). However, during the S5-S6 stages, the carpellary lateral vascular bundles gradually deformed and assumed an irregular shape in the fruit maturity stage (Figs. 16F to H).

### Anatomical features and development of blueberry seeds

**Fig. 17:**
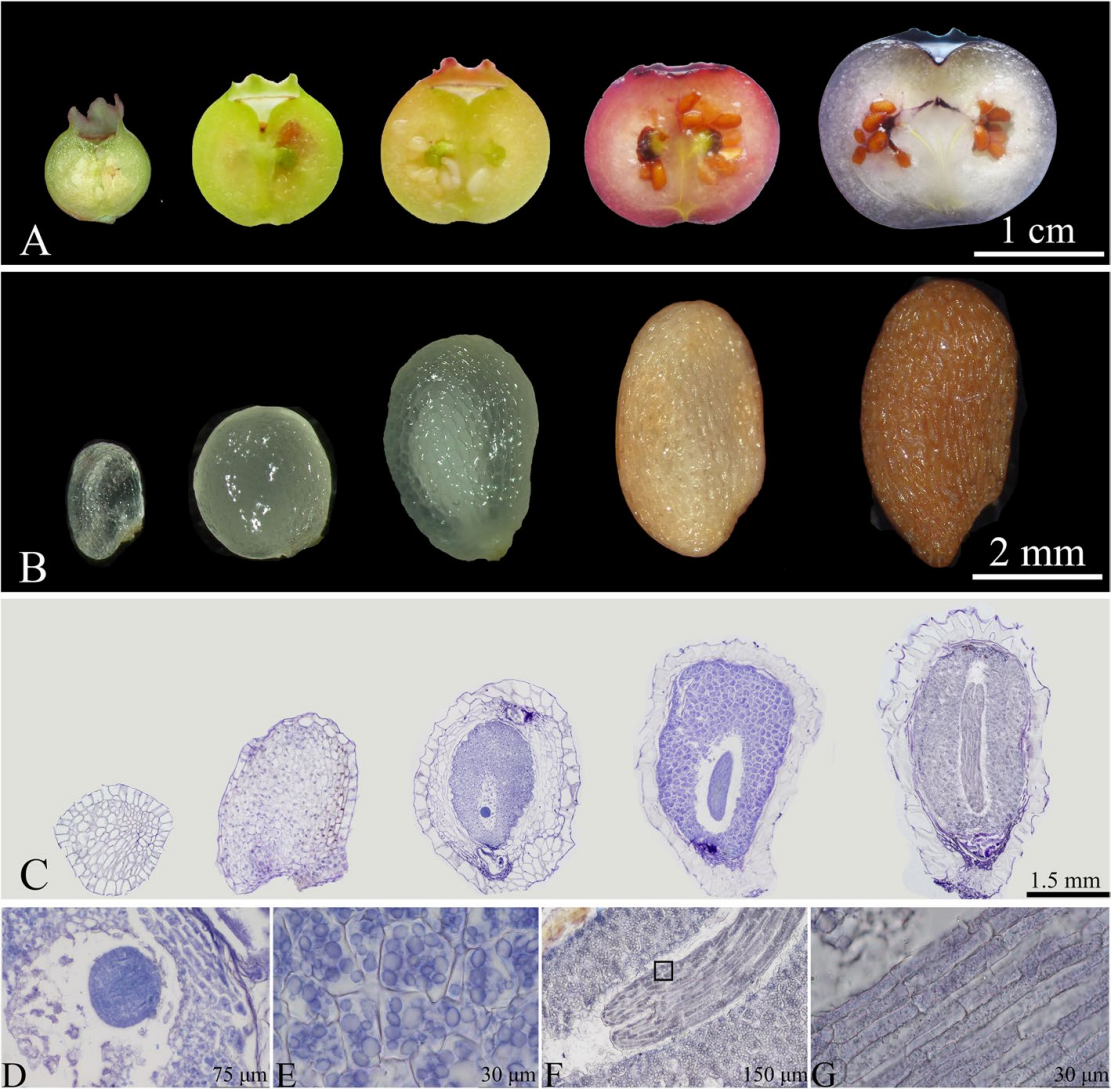
Developmental dynamics of blueberry seeds. **A** Longitudinal section of the blueberry fruit; **B** Equatorial view of the seed; **C** Longitudinal paraffin section of the seed; **D** Globular embryo; **E** endosperm cells; **F** "Y"-shaped cotyledons; **G** Enlarged view of the area within the black frame in Fig. 17F

The development of blueberry seeds was generally synchronized with the fruit development process (Fig. 17A). Mature blueberry seeds were light brown, 4.13 ± 0.42 mm long, 2.2 ± 0.14 mm wide, and elongated in shape, with a reticulate pattern that was evenly distributed. The reticular pattern was characterized by wide, raised walls and the mesh was densely packed. Each fruit contained about 50 to 60 seeds (Fig. 17B). During the seed development process, their color changed from white to pale yellow and eventually to light brown. The seed comprised the seed coat, endosperm, and embryo (Fig. 17C). The endosperm was white, smooth, and plump, occupying most of the seed volume, enveloping the embryo with varying thickness, and developed and matured ahead of the embryo maturation (Fig. 17E). The embryo comprised the embryonal axis, radicle, and cotyledons (Figs. 17F to G). The embryo, situated at the micropylar end of the endosperm, was surrounded by the micropylar endosperm, outer endosperm, and a thick-walled seed coat. The embryonal axis, radicle, and cotyledons were spatially separated from the endosperm based on the connections between the embryo, endosperm, and their respective parts. The embryo entered the globular embryo stage during the first rapid growth period of fruit development (Fig. 17D). The seed coat was light brown and hardened as the seed developed; the outermost layer of seed coat cells underwent lignification and secondary thickening. Pits in the cell wall formed tubular pit canals, eventually developing into palisade stone cells, with an inner layer of epidermal tissue present.

## Discussion

### Blueberry fruit development

Fruit growth and development is a complex process in which its shape, size, color, and quality undergo specific changes [19]. Many factors cause variations in fruit shape, such as varietal differences (genetic variations), plant nutritional levels, developmental and metabolic regulation by endogenous hormones, and environmental factors. In this study, fruits from the "Powderblue" blueberry cultivar had an average weight of 1.73 ± 0.17 g and a shape index of 0.91, categorized as elliptical, similar to findings by Medeiros et al. [20] in "Powderblue" blueberries. Fruit color is considered an evolutionary adaptation for increasing the probability of being discovered by dispersal agents, and its regulatory mechanisms are very complex [21]. Changes in pigmentation are markers for certain developmental stages and the physiological status of fruit, crucial for optimal storage and postharvest management [22]. The blueberry fruit color is mainly a result of anthocyanin biosynthesis and accumulation [10, 11]. Throughout the growth and development of "Powderblue" blueberry fruits, the color transitions from green to yellow to red, gradually deepening the red color until the fruit turns blue-black. This change in fruit color results from the varying expression of color-related genes and the joint action of environmental factors [23]. During the ripening process, most fruits change color drastically in the final stage of maturity. The blue color in blueberry fruits is considered an optimal standard for determining maturity and indicative of an optimal time for harvesting [24]. In the later stages of "Powderblue" blueberry fruit growth and development, a rapid transition from red to blue-black coloration is observed. Moreover, the change of fruit color to blue indicates that the fruit has matured and is ready for harvesting.

The growth curve represents the fruit's growth pattern after fertilization, which is also an important monitoring indicator for plant management in the field and choosing the appropriate harvest time. Fruit growth curves mainly exhibit either a single "S" curve or a double "S" curve pattern. For fruits with a single "S" growth curve, the growth rate shows a "slow-fast-slow" trend, as observed in fruit trees like pears [25] and apples [26]. Fruits with a double "S" curve typically exhibit two rapid growth and development periods during fruit growth, with the growth rate displaying a "slow-fast-slow-fast-slow" trend, as observed in peaches [27], strawberries [28], grapes [29], and other fruit trees. Extensive research has demonstrated that blueberry fruits exhibit a double "S" growth pattern during development [12, 30], which correspond to three distinct fruit development stages: the first stage, where a sharp increase in the size of the exocarp cells occurs; the second stage (also known as the lag phase), where the embryo and endosperm tissues develop rapidly; and the third stage, where a second rapid development of the exocarp cells occurs[31, 32]. Based on the results of our study, the single fruit weight, transverse diameter, and longitudinal diameter of "Powderblue" blueberries all followed a double "S" growth pattern, with the variation in the rate of single fruit weight increase being significant. In contrast, the transverse and longitudinal diameters changes during fruit development were less pronounced.

### Blueberry fruit cell development

Cell division and cell enlargement are considered the key determinants of fruit size and weight increase [33], where cell division determines the final number of cells, and cell enlargement determines the ultimate size of the cells. Therefore, the fruit's final size and weight are determined by the number and size of its cells. Cell divisions during fruit development mainly occur during the early stages of fruit development, determining the number of cells in the fruit and largely determining its final size [6]. Cell expansion and enlargement mainly take place in the middle and late stages of fruit development, increasing the fruit cell volume of fleshy fruits such as strawberries and cherries during the later stages of development [34]. It has been demonstrated that the rapid growth and development of many fleshy fruits is mainly through cell proliferation and expansion in the mesocarp [35]. Furthermore, cell expansion and enlargement take place concurrently with cell division in fruits; the cell number increase in the early stages lays the foundation for later expansion and enlargement, with more cells leading to more extensive enlargement and more pronounced increases in transverse and longitudinal diameters as well as single fruit weight. In this study, we found that cell division and cell enlargement occurred simultaneously during the growth and development process of "Powderblue" blueberries. The early stages of fruit growth and development were mainly characterized by increased cell division activities, resulting in a higher rate of increase in cell numbers. As the fruit developed, the cell number rate of increase gradually declined, with placental tissue cells exhibiting the most significant change. In the middle and late stages of "Powderblue" blueberry growth and development, the predominant feature was cell expansion and enlargement, with the rate of increase in single cell area in the middle and late stages being greater than that in the early stages. This was evident in the fruit flesh tissue cells, while the placental tissue cells and epidermal cells showed less pronounced changes.

Softening is an irreversible process that occurs in most fleshy fruits during maturation [36, 37]. Fruit softening is primarily caused by cell wall degradation [38–40]. As observed in this study, during the growth and development of "Powderblue" blueberries, the cell walls of the thin-walled cells in all tissues, except for the epidermal cells, undergo varying degrees of degradation from stage S4 onwards. The extent of cell wall degradation increases with the progression of the fruit development and its maturation, resulting in the continuous softening of the fruit's texture as a macroscopic trait. Brachysclereids, or stone cells, are widely distributed across the leaves and other organs of about 430 plant genera in 11 families in over 47 orders of angiosperms [41]. Due to their thick lignified secondary walls are considered to have a mechanical function, protecting soft tissues from mechanical damage or herbivory [42]. Although stone cells are widespread in most plant tissues throughout the plant body [43], most of our knowledge on stone cell functions comes from leaf stone cells, with little research on stone cells in plant tissues. During the S5 stage of "Powderblue" blueberry fruit growth and development, stone cells were observed in all fruit tissues except for the epidermal cells and seeds. These stone cells, which are brachysclereids, were similar in shape to the thin-walled tissue cells but had much thicker cell walls. The fruit flesh stone cells were mostly individually dispersed, whereas, in the placental tissue, they tended to cluster together. The number of stone cells increased as the fruit grew and developed. The epidermal cells do not rupture during fruit development, coinciding with the absence of stone cells within this tissue. Different degrees of rupture occur in cells from other tissues, and stone cells are also present in these tissues, emerging simultaneously or after the cells begin to rupture. The placental cells show the least degree of dissolution, and their stone cell content is significantly higher than in other tissues. The rupture of the fruit cell walls results in the inability of the flesh tissue to maintain structural integrity. However, the presence of stone cells, to a certain extent, supports and maintains the structure of the fruit flesh.

### Blueberry seed development

The fruits of the mother plant serve as the seed carrier, with continuous embryo growth, cellularization of the endosperm, and formation of the seed coat taking place in the seeds as the fruit develops [44–46]. The development of blueberry seeds was largely synchronized with the fruit development process. As seeds developed, the fruit tissues underwent a combined process of cell division and cell enlargement, promoting fruit growth. During the development of "Powderblue" blueberry seeds, the endosperm matured before the embryo, presenting as white, and was spatially separated from the embryo, with the seed coat gradually lignifying into a hard shell. The fruit ripened when seeds matured, and the surrounding tissues dried out or matured, facilitating seed dispersal [47]. The "seed control" hypothesis suggests that seeds communicate with their surrounding tissues via hormones and then promote fruit growth and development initially through cell division activation and then through cell expansion and enlargement [48]. In all fruits, the tissues surrounding the seeds undergo changes in color and cell wall components, leading to cracking or softening [49]. In "Powderblue" blueberries, which are classified as fleshy fruits, the tissues surrounding the seeds exhibited cell wall rupturing, leading to fruit softening when maturation was reached.

Seed size affects the growth and adaptability of plants and determines the seed yield of crops. Seed sizes vary widely among plant species, and this wide variation has been described as an important adaptive trait. How plants determine seed size is an important problem in developmental biology. Some researchers believe that the integument or seed coat determines the upper limit of the final seed size [50], and the integument affects the final seed size by influencing and determining the volume of the cavity for embryo and endosperm development. For example, the DA1 pathway controls the seed size of Arabidopsis by regulating cell proliferation in the integument[51, 52]. It has also been shown that endosperm growth is decisive for seed size, and in Arabidopsis, multiple factors have been shown to control seed size by regulating endosperm growth [53, 54]. The "Powderblue" seed of mature blueberry is light brown, with a length of (4.13 ± 0.42) mm and a width of (2.2 ± 0.14) mm, and the shape is oblong, similar to that of general blueberry seeds [55]. Understanding the mechanism of seed size control has become an important research field in plant science. In the development process of blueberry "Powderblue" seeds, the endosperm mature before the embryo, the endosperm is white, the embryo space is separated from the endosperm, and the seed coat gradually lignified into a hard shell. Therefore, the blueberry seed size may be caused by the joint action of the endosperm and seed coat, which needs further investigation.

## Conclusion

Throughout the growth and development of "Powderblue" blueberry fruits, the single fruit weight, transverse diameter, and longitudinal diameter of "Powderblue" blueberries all followed a double "S" growth pattern, with the variation in the rate of single fruit weight increase being significant. In contrast, the transverse and longitudinal diameters changes during fruit development were less pronounced, and the color transitions from green to yellow to red, gradually deepening the red color until the fruit turns blue-black. during the growth and development of "Powderblue" blueberries, the cell walls of the thin-walled cells in all tissues, except for the epidermal cells, undergo varying degrees of degradation from stage S4 onwards. During the S5 stage of "Powderblue" blueberry fruit growth and development, stone cells were observed in all fruit tissues except for the epidermal cells and seeds.Preliminary observations indicate a certain correlation between the degree of rupture in the blueberry fruit cells and the relative content of stone cells in the respective tissues. The development of "Powderblue" blueberry seeds was in sync with that of the fruit; when seed development was completed, the fruit ripened.The "Powderblue" seeds mainly consisted of the seed coat, endosperm, and embryo. The endosperm, occupying the vast majority of the seed volume, comprised both the chalazal and outer endosperm.

## Data Availability

No datasets were generated or analysed during the current study.
